# Tumor abnormal protein as a promising biomarker for screening solid malignancies and monitoring recurrence and metastasis

**DOI:** 10.3389/fonc.2023.1290791

**Published:** 2023-12-05

**Authors:** Zhihui Zhang, Changjun Tian, Yuexuan Liu, Lin Zhang, Han Sun, Siqi He, Yujia Liu, Hui Fan, Yongsheng Zhang, Mingxin Gao, Shuhua Wu

**Affiliations:** ^1^ Department of Geriatrics, The Second Affiliated Hospital of Soochow University, Suzhou, Jiangsu, China; ^2^ Department of General Medicine, The Second Affiliated Hospital of Soochow University, Suzhou, Jiangsu, China; ^3^ Department of Pathology, The Second Affiliated Hospital of Soochow University, Suzhou, Jiangsu, China

**Keywords:** tumor abnormal protein (TAP), solid malignancies, tumor biomarkers, diagnostic, recurrence, metastasis, prognosis

## Abstract

**Background:**

Tumor abnormal protein (TAP), the sugar chain protein released by tumor cells during metabolism, allows the development of a technique that exploits aggregated tumor-associated abnormal sugar chain signals in diagnosing malignancies. Clinically, we have found that TAP detection can well predict some malignancies, but several physicians have not paid attention, and related studies have been minimal.

**Methods:**

We evaluated TAP’s ability to distinguish between malignancies and benign diseases by receiver operating characteristic (ROC) curve analysis and studied the possibility of monitoring malignancy progression by evaluating TAP levels in follow-up. We used Kaplan-Meier survival curves and Cox proportional hazard regression models to investigate the relationship between TAP and prognosis.

**Results:**

TAP levels were higher in whole solid malignancies and every type of solid malignancy than in benign patients. ROC curve analysis showed that TAP levels aid in distinguishing between malignancies and benign diseases. TAP levels decreased in patients with complete remission (CR) after treatment and increased in patients with relapse from CR. Patients with metastases had higher TAP levels than non-CR patients without metastases. There was no difference in overall survival among patients with different TAP levels, and multivariate analysis suggested that TAP was not an independent risk factor for solid malignancies.

**Conclusion:**

TAP is an effective screening biomarker for many solid malignancies that can be used to monitor the progression of malignancies but not to prognosticate.

## 1 Introduction

Cancer is the leading cause of death worldwide and a substantial obstacle to increasing life expectancy (1). According to estimates from the World Health Organization in 2019, cancer is the first or second leading cause of death before age 70 in 112 of 183 countries (2). Earlier diagnosis of cancer is emerging as a revolution in increasing life expectancy among the general population for which a convenient, sensitive, and broad-spectrum screening method is needed (3, 4). Tumor abnormal protein (TAP), also known as tumor abnormal sugar chain protein, is a complex of glycoprotein and calcium-histone released by tumor cells during metabolism (5, 6) that aggregates tumor signals by reflecting a variety of tumor-associated abnormal sugar chains in peripheral blood (7). Many reports (8–10) have demonstrated that TAP is closely related to various tumors. For example, Ma et al. (8) found that TAP combined with transvaginal ultrasound was more accurate for diagnosing early endometrial cancer. Zhang et al. (9) reported that TAP was helpful in the initial diagnosis of bladder cancer. In addition, Li et al. (10) showed that the overall survival rates of patients with low TAP expression in pancreatic, gallbladder, bile duct, and liver cancers were significantly elevated compared with those of patients with high TAP expression. Therefore, TAP detection is a promising screening method for various solid malignant tumors.

For diagnosed malignant tumors, monitoring disease progression, recurrence and metastasis, and timely treatment are essential to improve the overall survival of patients (11, 12). Currently, the commonly used monitoring methods for malignant tumors are high-resolution computed tomography or magnetic resonance imaging combined with conventional tumor biomarkers (13–15). However, it is difficult to detect changes in the number of minimal malignant tumor cells with imaging methods (13). Therefore, a sensitive monitoring method that can detect a slight increase in malignant tumor cells earlier is important. TAP is present at high concentrations with aggregated tumor signals (7), and it may be possible to identify a small increase in malignant tumor cells.

There are few studies on TAP in the diagnosis of solid malignant tumors. It is still unclear whether TAP can be used to screen overall solid malignant tumors and monitor malignant tumor progression. Therefore, we studied TAP for distinguishing malignancies and monitoring metastasis and postoperative recurrence, hoping to find a sensitive biomarker for diagnosing and monitoring malignancies.

## 2 Materials and methods

### 2.1. Research population

The population in this study was inpatients of the Department of General Practice in the Second Affiliated Hospital of Soochow University from October 2017 to May 2022. Inclusion criteria for malignancy patients: 1) pathological examination confirmed the diagnosis of solid malignancies, 2) TAP levels were detected, 3) informed consent was signed at admission. Exclusion criteria for malignancy patients: 1) the primary sites of the tumor were unclear, 2) sarcoma and hematological malignancies, 3) refused to participate in this study during follow-up. Inclusion criteria for benign disease patients: 1) TAP detection was performed, 2) imaging detection and tumor biomarkers detection were used to exclude malignancies. Exclusion criteria for benign disease patients: 1) precancerous lesions, 2) unclear diagnosis. There were 901 inpatients, including 544 benign diseases, 357 malignancies, 549 male patients, 352 female patients, 294 nonelderly adult patients, and 607 elderly patients. Among benign disease patients, 198 were nonelderly, 346 were elderly, 329 were male, and 215 were female. Among malignancy patients, 96 were nonelderly, 261 were elderly, 220 were male, and 137 were female. Of 357 patients with malignancies, 204 did not begin treatment (initial diagnosis), 133 had received treatment, 70 were in complete remission (CR) after treatment, 107 with tumors had no metastasis, and 154 with tumors had metastasis.

### 2.2 Inpatients

According to the diagnostic criteria of the World Health Organization (WHO), we assigned inpatients who had received the TAP test to a benign disease group and a malignant tumor group, which excluded the precancerous lesion patients. The diagnosis of malignancies depends on pathological examination. The nasopharynx, oral cavity thyroid, breast, lung, liver, kidney, pancreas, gallbladder, bile duct, uterus, ovary, bladder and prostate tumor tissues were collected by needle biopsy or surgical resection. The tumor tissues in the throat, lung, esophagus, stomach, duodenum, jejunum, ileum, and colorectal were collected by laryngoscope, bronchoscopy, gastroscopy and enteroscopy. Exfoliated tumor cells were detected in alveolar lavage fluid, pleural effusion, peritoneal effusion, pelvic effusion and urine. All benign disease patients were excluded from malignancies by high-resolution computerized tomography (CT) or magnetic resonance imaging (MRI), supplemented by tumor biomarkers and combined with clinical symptoms and signs.

### 2.3 TAP detection

TAP detection is a standard clinical test in the Second Affiliated Hospital of Soochow University. Peripheral blood was obtained from fingertips with three drops of TAP reagent (Zhejiang Zijing Biotechnology Co. Ltd.) on the blood smear, and a coagulation staining reaction was observed until aggregated particles appeared. Two pathologists measured the TAP aggregated particles with a 40x objective lens.

### 2.4 Follow-up

The survival data of 50 patients who were newly diagnosed and receiving standard treatment were obtained by follow-up. We further collected the relevant parameters of these patients, and compared the parameters of surviving and nonsurviving patients. Parameters with a *P* value less than 0.2 were included in the multivariate Cox proportional hazard regression model to explore the risk factors affecting the overall survival of patients with solid malignant tumors. Tumor biomarkers include alpha-fetoprotein (AFP), carcinoembryonic antigen (CEA), carbohydrate antigen 125 (CA125), carbohydrate antigen 153 (CA153), carbohydrate antigen 199 (CA199), carbohydrate antigen 242 (CA242), total prostate, specific antigen (t-PSA), free prostate, specific antigen (f-PSA), cytokeratin 19 fragment (CYFRA21-1), neuron-specific enolase (NES), and gastrin-releasing peptide precursor (ProGRP). If one or more tumor biomarkers were greater than the reference range, the result could be considered positive.

### 2.5 Statistical methods

We utilized the SPSS 25.0 software package (SPSS, USA) for the statistical analysis. First, we used the median to represent continuous variables with skewed distribution. Second, we performed Mann-Whitney’s U test for comparing continuous variables and Pearson Chi-square analysis or Fisher’s exact test for categorical variables. Third, we used the receiver operating characteristic (ROC) curve and the area under the ROC curve (AUC) to test the ability of TAP levels to distinguish patients. Fourthly, we generated Kaplan-Meier curves to describe overall survival. Finally, we used a multivariate Cox proportional risk regression model to find independent risk factors affecting the overall survival of patients with solid malignant tumors. It was statistically significant for all statistics that a two-tailed *P* value was less than 0.05.

## 3 Results

### 3.1. The levels of TAP in inpatients with malignancies

**Figure 1:**
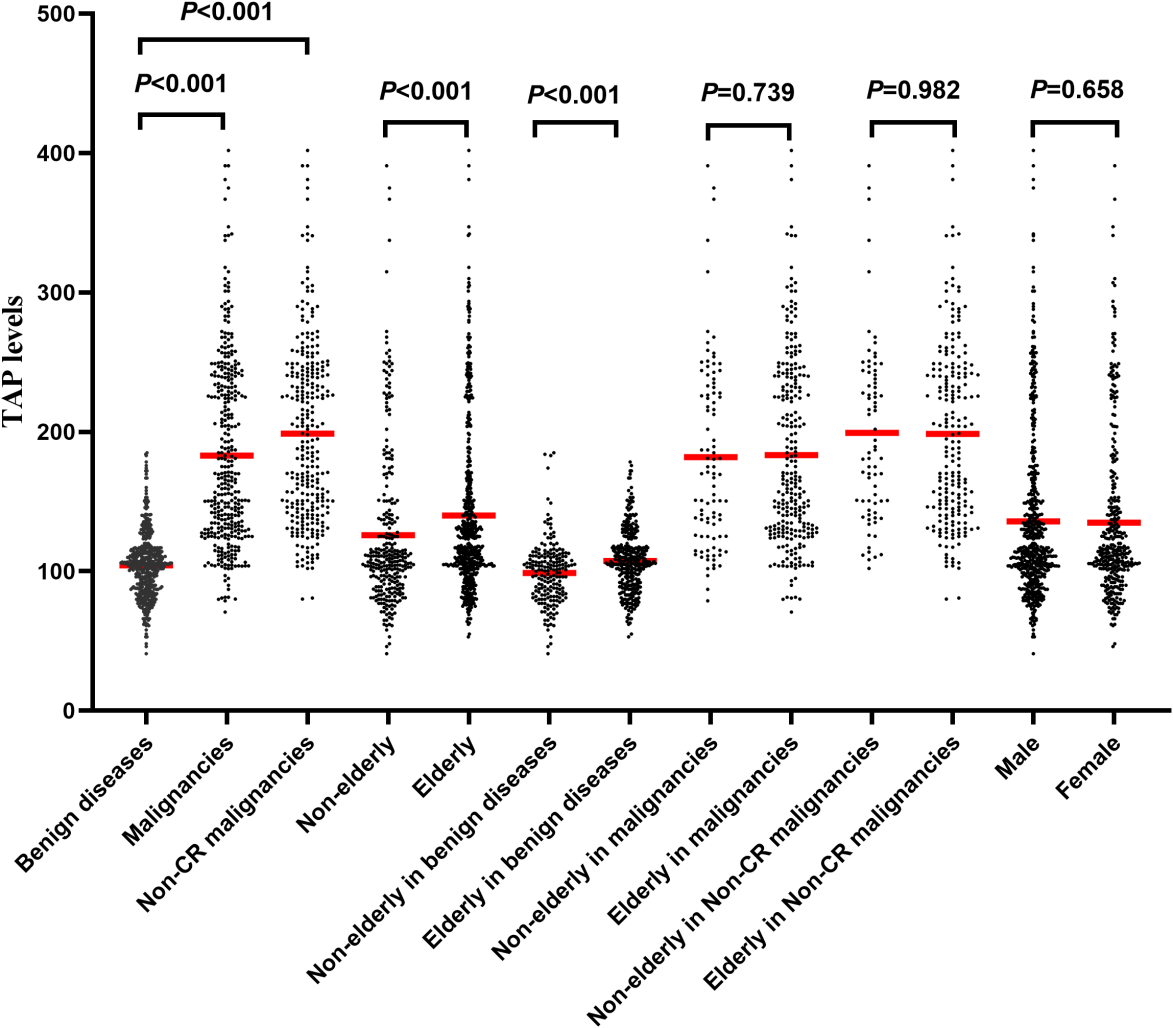
The levels of TAP in malignant and benign disease patients.

The levels of TAP in patients with malignancies [median 165.64 (range 70.72-402)] were significantly higher than those in patients with benign disease [median 104.81 (range 41-185), *P<*0.001, **Figure 1**]. After excluding patients with CR, we found that the levels of TAP in non-CR patients with malignancies [median 189.53 (range 80.05-402)] were significantly higher than those in patients with benign disease [median 104.81 (range 41-185), *P<*0.001, **Figure 1**]. The TAP levels of elderly patients were significantly higher than those of nonelderly patients (*P*<0.001, **Figure 1**). Among the benign disease patients, the TAP levels of elderly patients were significantly higher than those of nonelderly patients (*P*<0.001, **Figure 1**). Among the overall and non-CR malignancy patients, there was no significant difference in TAP levels between elderly and nonelderly patients (*P*=0.739 and 0.982, **Figure 1**). There was no significant difference in TAP levels between male and female patients among prevalent benign disease and malignancy patients (*P*=0.658, *P*=0.677, and *P*=0.977, **Figure 1**).

### 3.2 Distinguishing TAP levels between malignant and benign diseases

**Figure 2:**
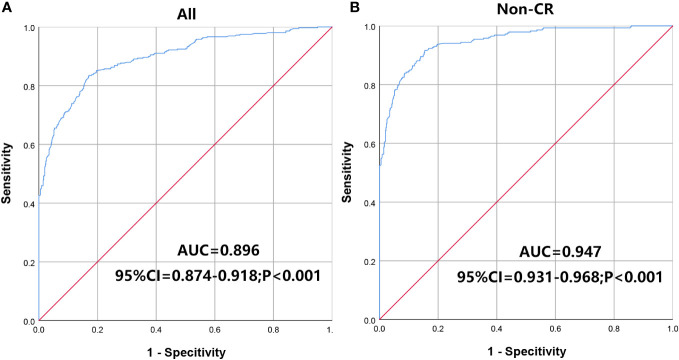
ROC curve analysis to divide the patients into malignant and benign diseases. **(A)** all malignant patients; **(B)** Non-CR malignant patients. AUC, area under the ROC curve.

ROC curve analysis was performed based on the TAP levels of patients with malignancies and benign diseases. We found that TAP levels were significantly different between malignant and benign conditions (AUC=0.896, 95% confidence interval (CI)=0.874-0.918, *P*<0.001, **Figure 2**). When the cutoff value of the TAP level was 123.20, the Youden index (sensitivity + specificity -1) was the largest with the discrimination ability of malignancies. For non-CR malignancies compared with benign diseases, the AUC was 0.947 (95% CI=0.931-0.968, *P*<0.001, cutoff value=125.02, **Figure 2**).

### 3.3 Clinical relevance of TAP levels and tumor biomarkers and metastasis

According to the median TAP level, we assigned initially diagnosed patients to two groups: a high TAP level group and a low TAP level group. The positive rates of traditional tumor biomarkers and tumor metastasis in the high TAP-level group were higher than those in the TAP low-level group (*P*=0.039 and *P*<0.001, respectively, **Table 1**). There were no significant differences in age or sex between the two groups (*P*=0.623 and *P*=0.469, respectively, **Table 1**).

**Table 1:** Clinical differences of patients with different TAP levels.

Characteristics	TAP low level group (n=102)	TAP high level group (n=102)	*P*
**Age (year)**	70(30-94)	70(27-100)	0.623
**Sex**			0.469
*Female*	35	41	
*Male*	67	61	
**Tumor biomarkers**			0.039
*Positive*	56	72	
*Negative*	43	29	
**Metastatic**			
*Yes*	33	61	<0.001
*No*	65	33	

TAP, tumor abnormal protein.

### 3.4 The prognosis of patients with different TAP levels

**Figure 3:**
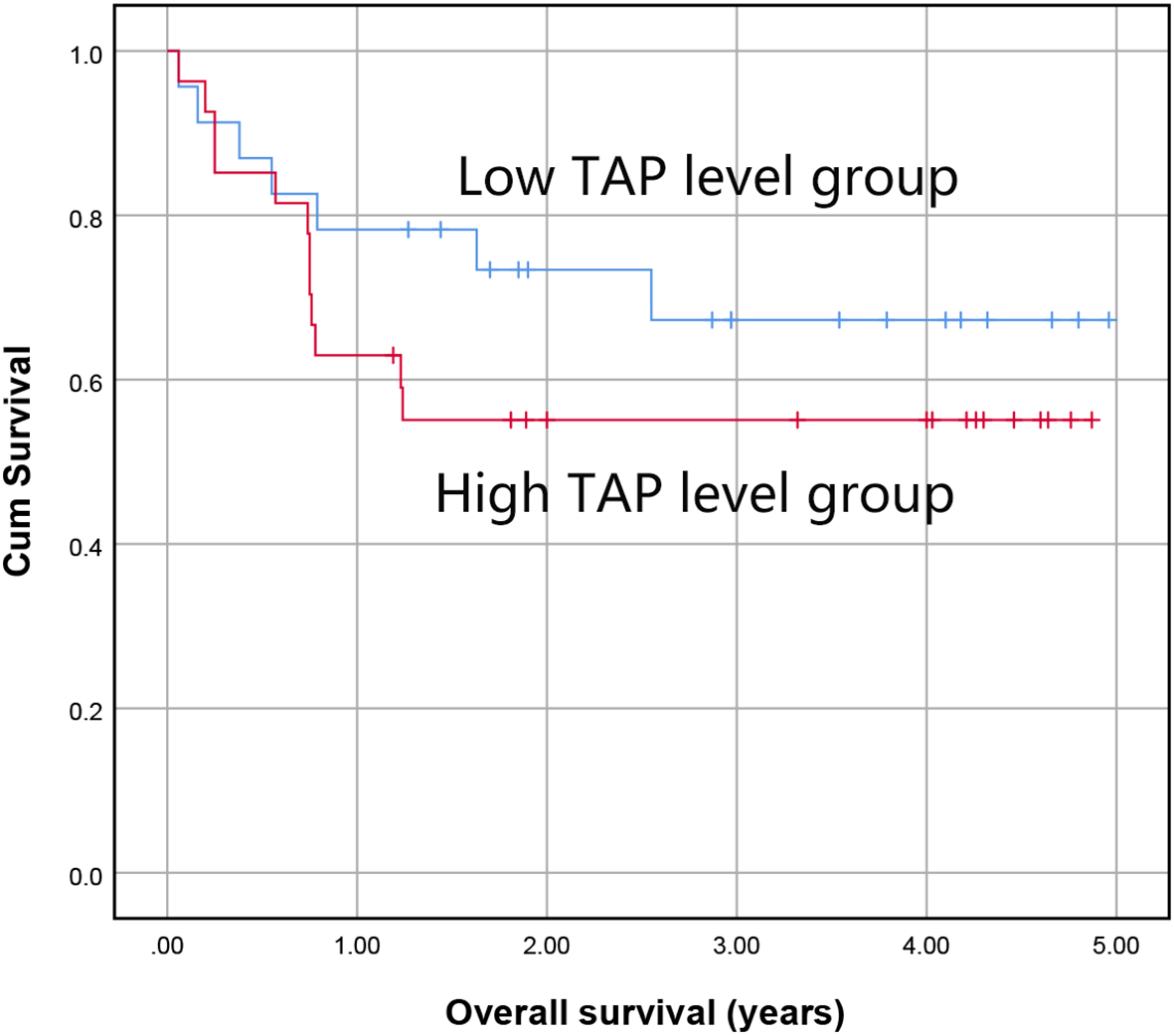
The impact of TAP levels on overall survival of malignancies.

We followed up on the overall survival time of patients receiving standard treatment. The patients with malignant tumors were categorized into a high TAP group and a low TAP group by the median TAP level, and Kaplan-Meier curve analysis was performed. There was a trend of lower survival time in the high TAP group, but the difference was not statistically significant (*P*=0.306, **Figure 3**).

### 3.5 Independent risk factors affecting overall survival in patients

We compared the parameters of living and dead patients and found that there were significant differences in age, tumor biomarkers, hemoglobin (HB), and serum sodium (Na) levels (*P*<0.05, **Table 2**). Parameters with a *P* value less than 0.2 (including age, sex, TAP, tumor biomarkers, HB, white blood cell (WBC), serum creatinine (Scr), triglyceride (TG), and Na) were included in the multivariate Cox proportional hazard regression model to explore the independent risk factors affecting the overall survival of solid malignant tumors. TAP was not associated with the survival of solid malignant tumors (*P*=0.434, **Table 3**). Female sex, abnormal tumor markers, and low hemoglobin levels were independent risk factors for the overall survival of solid malignant tumors (*P*=0.013, 0.003, <0.001, respectively, **Table 3**).

**Table 2:** Parameter differences between alive and dead patients.

Characteristics	Alive(n=31)	Dead(n=19)	*P*
**Age** (year)	54(27-81)	70(48-88)	0.003
**Sex**			0.077
*Female*	13	9	
*Male*	18	10	
**TAP** (μm^2^)	185.15(108.42-381)	229(136-402)	0.064
**Tumor biomarkers**			0.041
*Positive*	15	15	
*Negative*	16	4	
**Smoking**			0.722
*Yes*	7	3	
*No*	24	16	
**Alcohol drinking**			1
*Yes*	5	3	
*No*	26	16	
**Family history of cancer**			1
*Yes*	1	1	
*No*	30	18	
**HB (**×10^9^/L)	133(72-167)	101(62-127)	<0.001
**WBC (**×10^9^/L)	6.4(3.8-10.2)	7.7(3-14.3)	0.171
**PLT (**×10^9^/L)	248(139-531)	196(129-453)	0.299
**ALT (**U/L)	15(6-200)	14(6-338)	0.787
**AST (**U/L)	18(11-174)	22(10-389)	0.441
**Scr** (μmoI/L)	62(39-94)	53(40-106)	0.055
**TC (**mmol/L)	4.41(2.54-7.36)	4.15(1.92-6.54)	0.385
**TG (**mmol/L)	1.12(0.35-3.65)	1.35(0.68-8.67)	0.144
**K (**mmol/L)	3.93(2.79-4.47)	4.2(2.81-4.98)	0.219
**Na (**mmol/L)	141.3(137.8-145.3)	138.9(124.3-147.4)	0.002

TAP, tumor abnormal protein; HB, hemoglobin; WBC, white blood cell; PLT, platelet; ALT, alanine transaminase; AST, aspartate transaminase; Scr, serum creatinine; TC, total cholesterol; TG, triglyceride; K, serum potassium; Na, serum sodium.

**Table 3:** Multivariate Cox proportional risk regression model for overall survival of solid malignant tumors.

Factors	HR	95%CI	*P*
Age	1.050	0.994-1.109	0.084
Sex	0.126	0.025-0.641	**0.013**
TAP	0.997	0.989-1.005	0.434
Tumor biomarkers	8.366	2.090-33.488	**0.003**
HB	0.937	0.905-0.969	**<0.001**
WBC	1.028	0.833-1.268	0.799
Scr	0.994	0.956-1.033	0.755
TG	1.022	0.744-1.405	0.892
Na	0.929	0.806-1.072	0.314

HR, hazard ratio; CI, confidence interval; TAP, tumor abnormal protein; HB, hemoglobin; WBC, white blood cell; Scr, serum creatinine; TG, triglycerid; Na, serum sodium.

### 3.6 TAP levels in different types of malignancies

Malignancy patients included 7 bladder cancers, 7 bile duct cancers, 10 gallbladder cancers, 54 lung cancers, 15 liver cancers, 8 cervical cancers, 18 thyroid cancers, 44 colon cancers, 5 ovarian cancers, 30 prostate cancers, 13 breast cancers, 7 esophagus cancers, 54 gastric cancers, 16 pancreatic cancers, 33 rectal cancers, 19 multiple cancers, and 17 other cancers (included 1 skin cancer, 1 ureter cancer, 1 nasopharynx cancer, 1 pancreatic duodenal carcinoma, 1 neuroblastoma, 2 kidney cancers, 2 oral cancers, 4 duodenal cancers and 4 endometrial cancers).

**Figure 4:**
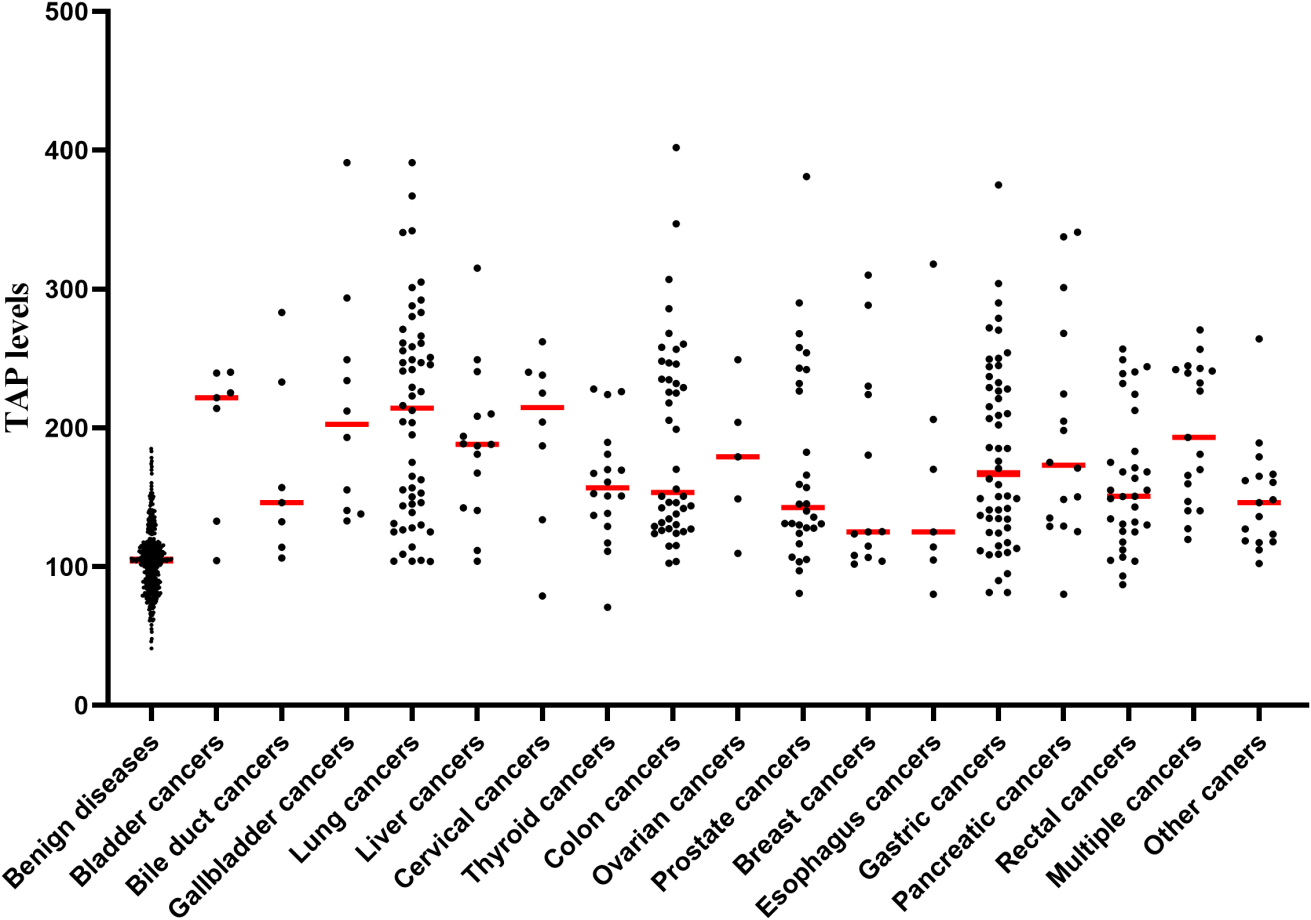
TAP levels in different malignancies.

By comparing the TAP levels between patients with different tumors and patients with benign diseases, we found that all malignant tumors’ TAP levels were higher than those of benign conditions. (*P*<0.05, **Figure 4**). Furthermore, comparing non-CR patients with benign patients further indicated that TAP levels were higher in all patients with malignant tumors than in benign diseases. There was no significant difference in TAP levels between different malignant tumors.

### 3.7 TAP levels vary before and after tumor removal

**Figure 5:**
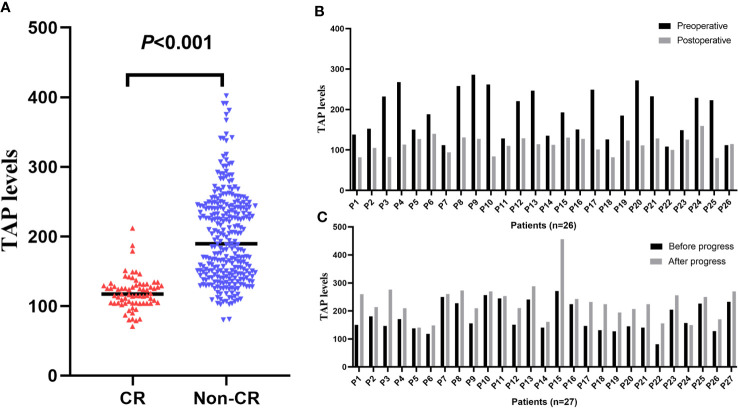
TAP levels vary with malignancy status. **(A)** The TAP levels of CR and non-CR patients; **(B)** Following up the patients from preoperative to post-operative; **(C)** Following up the progress of patients.

The TAP levels of CR tumor patients were lower than those of non-CR patients (*P*<0.001, **Figure 5**). By following up with the patients, we found that the TAP levels postoperation were significantly lower than those before the operation (*P*<0.001, **Figure 5**) and increased significantly after disease progression (*P*=0.001, **Figure 5**).

### 3.8 Distinguishing TAP levels on recurrence and CR after the operation

**Figure 6:**
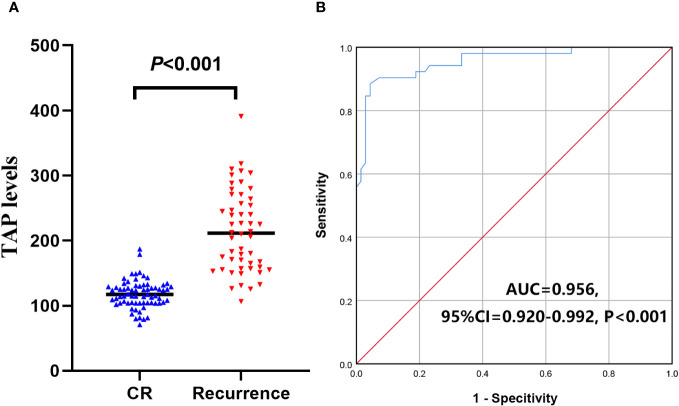
TAP levels divide the patients into CR and recurrence. **(A)** The TAP levels in CR and recurrence malignancies; **(B)** TAP level distinguish recurrence from CR.

The TAP levels of patients with CR were higher than those of patients with benign disease, which suggested that patients with CR after treatment still have a risk of recurrence. Compared with CR patients, the levels of TAP were significantly higher in patients with recurrence (*P*<0.001, **Figure 6**). ROC curve analysis indicated that the TAP level could distinguish recurrence from CR (AUC=0.956, 95% CI=0.920-0.992, *P*<0.001, **Figure 6**). When the TAP level was higher than 149.74, the CR patients might have had a recurrence.

### 3.9 Distinguishing TAP levels between metastatic and non-CR patients

**Figure 7:**
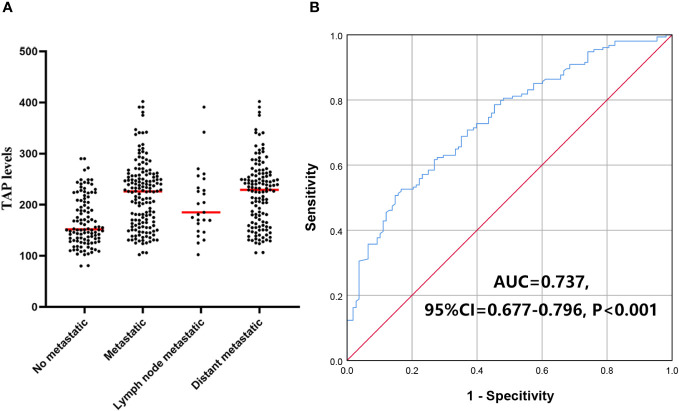
TAP levels divide the non-CR malignancies into metastatic and not. **(A)** The TAP levels in patients with and without metastatic; **(B)** TAP level distinguish metastasis and not.

Compared with non-CR patients without metastatic malignancies, TAP levels were significantly higher in patients with metastatic malignancies. TAP levels in patients with distant metastasis and lymph node metastasis were higher than those in patients with malignancies without metastasis (*P*=0.005 and *P*<0.001, respectively). TAP levels in malignancies with distant and lymph node metastasis were not significantly different (*P*=0.126). ROC curve analysis showed that TAP levels significantly distinguished between malignancies with no metastasis and metastasis (AUC=0.737, 95% CI=0.677-0.796, *P*<0.001, **Figure 7**), and the TAP cutoff value was 224.36. Above this, non-CR patients may have metastasized.

## 4 Discussion

Clinically, molecular biomarkers of malignancies with higher specificity make cancer diagnosis more precise (16, 17). In the case of population screening for an earlier diagnosis, broad-spectrum and sensitive biomarkers are more critical. TAP detection exploits aggregated tumor-associated abnormal sugar chain signals in diagnosing malignancies. Glycosylation, a hallmark of cancer, can produce tumor glycans or glycoproteins (18–21). Abnormal glycosylation due to cellular and metabolic changes leads to aberrant expression of glycans in the membrane, which initiates the malignant transformation of cells (18). Abnormal glycosylated proteins are regulators of the malignant phenotype of cancer cells (19). Altered glycosylation enables tumor cells to evade immune surveillance mechanisms (20). The cancer genome atlas has identified changes in the expression of glycosylation-specific genes associated with cancer progression (21). As an expected change in cancers, protein glycosylation may be a broad-spectrum biomarker for cancer surveillance.

TAP detection is a method to evaluate patients’ peripheral blood glycoprotein levels. Our study found a significant increase in the TAP levels of solid malignancies. Excluding the malignancies with CR after treatment, the TAP levels of non-CR malignancies were significantly different from those of benign diseases. ROC curve analysis revealed that TAP levels distinguished solid malignancies from benign tumors. TAP levels were elevated in overall malignancies and each type of solid malignancy involved in this study, indicating that TAP levels have diagnostic efficacy for various malignancies. However, Kaplan-Meier survival analysis and the Cox proportional hazard regression model revealed that TAP was not associated with the overall survival of patients with solid malignant tumors. We did not find the difference in TAP levels in different malignant tumors. As mentioned above, glycans are markers of malignant transformation of cells, and glycoprotein abnormalities may be a common feature of malignant cells. Currently, research on tumor biomarkers is becoming increasingly precise and individualized, but research on universal biomarkers of solid malignant tumors is minimal (22, 23). As a broad-spectrum screening method for solid malignant tumors, TAP may play an important role in population screening.

Interestingly, we found that the TAP levels in elderly patients were higher than TAP levels of nonelderly patients among those with benign disease; however, there was no significant difference in patients with solid malignancies. In the body of a human, cell cancer and clearing occur consistently (24, 25). The immune system can quickly identify and clear malignant cells; however, the ability of elderly patients in this regard is weakened (26). In elderly patients with nonmalignant tumors, there may be a small number of malignant cells in the body, and TAP levels can reflect slight differences, revealing the sensitivity of TAP. However, there are many malignant cells in malignant patients, and the difference caused by age is relatively small. Therefore, there is no significant difference in TAP levels between elderly and nonelderly malignancy patients.

Next, we wanted to determine whether we could track TAP level changes with tumor status to monitor malignancy. Therefore, we further analyzed the sensitivity of TAP in detecting malignancies. We found that the TAP levels of CR patients were significantly lower than those of non-CR patients, which indicated that TAP levels might vary with disease status. Thus, we followed up on malignancies and found that TAP levels in postoperative patients with solid malignancies were significantly lower than those in patients before surgery and increased after disease progression. This showed that TAP was a sensitive indicator for the dynamic monitoring of malignancies.

In patients with solid malignancies, monitoring disease progression is very important. Many patients achieve CR after surgical treatment but still have a high risk of recurrence (27). Currently, the commonly used monitoring methods are imaging examination and tumor biomarkers (28, 29). In this study, we compared the TAP level of malignancies with CR and recurrence and found that the TAP levels in patients with recurrence were significantly increased. ROC curve analysis found a TAP value greater than 149.74, indicating probable recurrence.

Previously, we compared the malignancies in the high-level and low-level TAP groups and found that the tumor metastasis rate was higher in the high-level group. Therefore, we further measured TAP levels in patients with metastatic and nonmetastatic malignancies, and we found that TAP levels were higher in patients with solid metastatic malignancies. ROC curve analysis revealed that the TAP level could distinguish metastatic and nonmetastatic malignant tumors. The malignant tumor may have metastasized when the TAP level was higher than 224.36. Tumor metastasis is an important contributor to the deaths of cancer patients (30). TAP detection is helpful for the early detection of tumor metastasis and can help improve patients’ overall survival time through early treatment adjustment.

## 5 Conclusion

TAP is a broad-spectrum screening tool for many solid malignant tumors that varies with malignant tumor status. It can be used to monitor the recurrence and metastasis of malignant tumors but is not associated with prognosis.

## Data availability statement

The original contributions presented in the study are included in the article/supplementary material. Further inquiries can be directed to the corresponding authors.

## Ethics statement

The studies involving humans were approved by Ethics Committee of the Second Affiliated Hospital of Soochow University. The studies were conducted in accordance with the local legislation and institutional requirements. The participants provided their written informed consent to participate in this study.

## Author contributions

SW: Project administration, Writing – original draft. ZZ: Investigation, Writing – original draft. CT: Writing – review & editing. YXL: Supervision, Writing – original draft. LZ: Data curation, Writing – original draft. HS: Validation, Writing – original draft. SH: Conceptualization, Writing – review & editing. YJL: Software, Writing – original draft. HF: Project administration, Writing – original draft. YZ: Methodology, Writing – original draft. MG: Investigation, Writing – original draft.
